# High-Throughput Targeted Repeat Element Bisulfite Sequencing (HT-TREBS): Genome-Wide DNA Methylation Analysis of IAP LTR Retrotransposon

**DOI:** 10.1371/journal.pone.0101683

**Published:** 2014-07-08

**Authors:** Muhammad B. Ekram, Joomyeong Kim

**Affiliations:** Department of Biological Sciences, Louisiana State University, Baton Rouge, Louisiana, United States of America; The Ohio State University, United States of America

## Abstract

In vertebrates, DNA methylation-mediated repression of retrotransposons is essential for the maintenance of genomic integrity. In the current study, we developed a technique termed HT-TREBS (High-Throughput Targeted Repeat Element Bisulfite Sequencing). This technique is designed to measure the DNA methylation levels of individual loci of any repeat families with next-generation sequencing approaches. To test the feasibility of HT-TREBS, we analyzed the DNA methylation levels of the IAP LTR family using a set of 12 different genomic DNA isolated from the brain, liver and kidney of 4 one-week-old littermates of the mouse strain C57BL/6N. This technique has successfully generated the CpG methylation data of 5,233 loci common in all the samples, representing more than 80% of the individual loci of the five targeted subtypes of the IAP LTR family. According to the results, approximately 5% of the IAP LTR loci have less than 80% CpG methylation levels with no genomic position preference. Further analyses of the IAP LTR loci also revealed the presence of extensive DNA methylation variations between different tissues and individuals. Overall, these data demonstrate the efficiency and robustness of the new technique, HT-TREBS, and also provide new insights regarding the genome-wide DNA methylation patterns of the IAP LTR repeat elements.

## Introduction

About half of the mammalian genome is comprised of repeat elements of different types [1],[2]. The bulk of these elements are retrotransposons and DNA transposons, making up 42% and 2–3% of the genome, respectively [3]. The ability of these repeat elements to move to new locations is inhibited by several epigenetic mechanisms of the host genome, including histone modifications and DNA methylation. The repeat elements are closely associated with the histone modifications H3K9me3 and H4K20me3 [4],[5]. Model organisms with mutations on the genes establishing these histone modifications tend to de-repress the transcription of repeat elements, confirming their repressive roles in the repeat elements [6]. Compared to the histone modifications, DNA methylation is a more permanent and stable epigenetic modification for the transcriptional repression of the repeat elements [7]. In mammals, genome-wide DNA methylation occurs at two different times of the development: early embryogenesis and gametogenesis [8]. The majority of repeat elements are similarly subject to these two waves of DNA methylation. DNMT3A is the primary enzyme repressing the repeat elements during germ cell development whereas DNMT1A is responsible for maintaining the established DNA methylation on the repeat elements during somatic cell replication [9]–[11]. Recent studies also indicated that the small non-coding RNAs, Piwi-interacting RNAs (piRNAs), play a critical role in repressing the transcription of the repeat elements during spermatogenesis [12],[13].

Although the majority of repeat elements are repressed by DNA methylation, a small fraction of these elements are also known to escape the DNA methylation-mediated repression. Two well-known cases include the mouse genomic loci *Agouti* and *Axin*. Both of these loci contain one type of retrotransposons, IAP (Intracisternal A Particle), and their LTRs (Long Terminal Repeats) are partially repressed by DNA methylation. Furthermore, the methylation levels of these two IAP LTR are variable between individual mice with visible phenotypic consequences, such as coat color variations for the viable yellow agouti (*A^vy^*) allele and tail kinkedness variations for the axin-fused kinky (*Axin^fused^*) allele, and are thus named ‘epialleles’ [14],[15]. Interestingly, the DNA methylation levels of these epialleles can be easily changed by environmental interventions during development [15],[16]. According to recent studies, additional mouse loci with retrotransposons, such as IAP and L1, also escape the DNA methylation-mediated repression with inter-individual variability [17]. In the case of humans, the repeat elements tend to be hypomethylated in cancer genomes [18],[19] although the functional relevance (driver or passenger) of the observed de-repression to cancer is debatable. It is thus clear that DNA methylation-mediated repression on the repeat elements is very critical for the maintenance of genomic integrity [20]–[23].

Despite the significant roles played by DNA methylation in the repeat elements, many important questions have not been addressed so far, such as what fraction of the repeat elements escape the DNA methylation-mediated repression and which individual repeat elements escape this repression. To address these questions, we developed and tested the feasibility of a new protocol named High-Throughput Targeted Repeat Element Bisulfite Sequencing (HT-TREBS). This new protocol is designed to provide genome-wide, single-base resolution, and highly enriched DNA methylation data of any subset of repeat elements. Using this protocol, we successfully analyzed the methylation status of individual loci of the mouse IAP LTR family. The results indicate that a minor fraction (about 5%) of IAP LTR are hypomethylated, and also that the methylation levels of majority of IAP LTR are variable between tissues and also between individuals.

## Results

### High-Throughput Targeted Repeat Element Bisulfite Sequencing (HT-TREBS)

**Figure 1:**
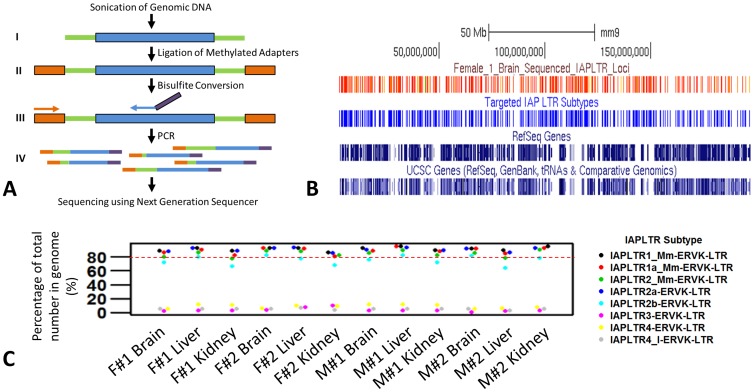
Scheme and efficiency of the High-Throughput Repeat Element Bisulfite Sequencing (HT-TREBS) technique using mouse IAP LTR elements. (A) Schematic of the HT-TREBS depicting the steps involved up to the run in a next-generation sequencer. The blue bars and the green bars in steps I-IV represent the IAP LTR sequences and the adjoining unique sequences respectively. The orange bars in steps II–IV represent the methylated Ion Torrent ‘A’ adaptor. The orange arrow in step III depicts the forward primer corresponding to the ‘A’ adaptor region while the blue arrow depicts the reverse primer corresponding to the chosen IAP LTR site. The reverse primer also has the Ion Torrent ‘P1’ adaptor sequence attached to it as depicted by the purple bar. Step IV shows that the PCR products generated from a single specific IAP LTR locus might have varying lengths because of the random lengths of the unique sequences (green bars) caused by sonication. (**B**) Efficiency of the HT-TREBS. The distribution of the sequenced IAP LTR loci (depicted in red) in chromosome 1 (mm9) of the Female #1 brain sample. The IAP LTR elements have been sequenced without any chromosome region bias. A comparison with the positions of the five targeted subtypes (depicted in blue) shows that the majority of the targeted loci have been sequenced. (**C**) Specificity of the HT-TREBS. Graph showing the percentage of the total number of different IAP LTR subtypes that have been sequenced in the all twelve samples. The dashed red line shows the level of 80%. The percentages of the total number of the five targeted IAP LTR subtypes (IAPLTR1, IAPLTR1a, IAPLTR2, IAPLTR2a, and IAPLTR2b) that have been sequenced are more than 80% in majority of the cases. The three non-targeted IAP LTR subtypes (IAPLTR3, IAPLTR4, and IAPLTR4_I), even though sequenced, were actually represented by a much smaller fraction of their total number in the genome (less than 12% in all the cases).

HT-TREBS is based on adaptations of the two high throughput bisulfite sequencing techniques: Reduced Representation Bisulfite Sequencing (RRBS) [24] and methylC-Seq [25]. In this scheme (**Fig. 1A**), one of the primers for a PCR step is specific to a set of targeted repeat elements, allowing the enrichment of only a subset of repeat elements for the subsequent analysis. The current study tested the feasibility of HT-TREBS by targeting 5 subtypes of the mouse IAP LTR retrotransposon family (IAPLTR1, IAPLTR1a, IAPLTR2, IAPLTR2a, and IAPLTR2b). In the entire study, individual LTR elements were considered separately even if they are one of the two LTRs of a full length IAP element. Likewise, the total number of IAP LTRs were counted and all the analysis were done by considering them as individual elements. The members of these subtypes (7,810 members in the mouse genome) share high levels of sequence identity within a small 24-bp long region of the LTR, thus the sequence of this region was used for designing a PCR primer for DNA methylation analyses as described below. In brief, a set of 12 different DNA samples was isolated from the brain, liver, and kidney (one representative tissue from each of the three germ layers) of four 1-week-old littermates (two females and two males) of the mouse strain C57BL/6N. Each isolated DNA was individually fractionated by sonication up to a predetermined length, end-repaired, and then ligated to custom-made next generation sequencing adaptors in which all the Cs have been methylated (**Fig. 1A** **and** **Material & Methods**). Since the presence of unique sequence is crucial for the success of this method, the desired length of the sonicated DNA was predetermined to be at 700 bp in length. Fragments of such length can have the full IAP LTR along with enough length of unique sequences flanking it. After a round of size selection to remove any undesirable short fragments and any excess adaptors, the adaptor-ligated DNA was treated with the bisulfite conversion method [26]. The bisulfite-converted DNA was amplified with PCR using a set of primers: a forward primer binding to the adaptor region and a reverse primer binding to the 24-bp small region of the IAP LTR. The amplified PCR product was size-selected for 250–300 bp using an agarose gel electrophoresis. The size range of the amplified product was chosen based on the optimum sequencing conditions of the Next-Generation Sequencing (NGS) machine used. The size-selected DNA was then sequenced using the NGS machine.

The sequence reads generated from each sample DNA were mapped to a custom reference genome containing bisulfite-converted IAP LTR sequences using the aligner Bowtie2 [27]. The mapped sequence reads were filtered by custom scripts to retain only those reads that mapped to an IAP LTR locus and also had at least 10 bases of flanking unique sequences to ensure the reads were sequenced from unique positions of the genome (**Materials &** **Methods**). The filtered sequence reads were subsequently analyzed using BiQAnalyzerHT to derive the methylation status of each IAP LTR [28]. In each sample, custom scripts were also employed to retain only those IAP LTR that had at least 3 CpG positions with the sequencing depth being at least 15 for each CpG position. The overall results of these bisulfite sequencing trials are summarized in **Table 1**. In 12 samples, an average of 1×10^6^ reads were successfully mapped to approximately 7,000 IAP LTR loci, representing, on average, >100X sequencing depth for each IAP LTR locus. The lengths of the sequence reads were long enough to analyze the methylation levels of an average of 7 CpG positions for a given IAP LTR. According to the results, the sequenced IAP LTR are evenly distributed over the entire lengths of all the chromosomes without any bias for any particular region of the chromosomes (**Fig. 1B** **and Fig. S1**). The sequenced IAP LTR also cover more than 80% of each of the initial 5 subtypes in almost all the samples based on the total number of each subtype in the mouse genome (**Fig. 1B&C**). The specificity of this targeted sequencing is further confirmed by the results that the non-targeted IAP LTR subtypes (IAPLTR3, IAPLTR4, and IAPLTR4_I) were represented by a much smaller fraction (less than 12% in all the cases). Overall, this demonstrates that HT-TREBS is an effective approach for high-throughput bisulfite sequencing for any class of repeat elements.

**Table 1:** Coverage and sequencing efficiency of the twelve samples sequenced by the HT-TREBS technique.

Sequenced sample	Number of mapped sequence reads that contain IAP LTR sequences and unique sequences *	Number of IAP LTR loci retained after filtering	Total number of CpG positions sequenced at depth of >15x	Average number of CpG positions sequenced per IAP LTR loci	Average coverage of the CpG positions
Female #1 Brain	992,155	6,525	49,856	7	123x
Female #1 Liver	787,780	6,403	48,543	7	100x
Female #1 Kidney	904,991	6,996	53,311	7	107x
Female #2 Brain	2,227,792	7,070	54,297	7	250x
Female #2 Liver	719,417	6,446	49,590	7	96x
Female #2 Kidney	926,852	7,055	54,005	7	109x
Male #1 Brain	712,576	6,875	52,614	7	89x
Male #1 Liver	963,293	6,680	51,221	7	125x
Male #1 Kidney	1,111,838	7,218	55,669	7	129x
Male #2 Brain	1,643,920	6,957	52,830	7	178x
Male #2 Liver	1,118,820	7,109	53,775	7	121x
Male #2 Kidney	659,375	7,914	43,497	6	71x

*Number of reads with IAP LTR sequences that mapped to the reference genome and had at least 10 bases of unique sequences.

### DNA methylation patterns of the IAP LTR retrotransposons

**Figure 2:**
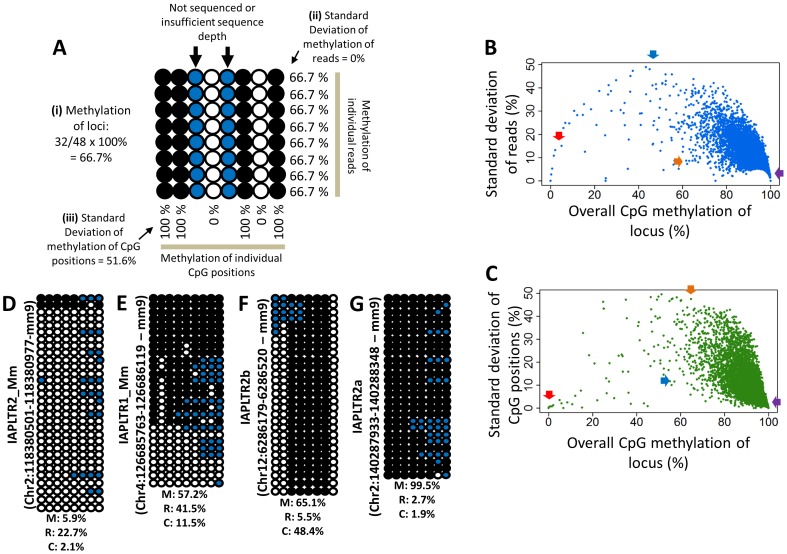
Depiction of the canonical methylation pattern of the IAP LTRs by ‘sprinkler plot’. (A) A hypothetical IAP LTR locus showing the calculation of the i) the overall CpG methylation of the locus, ii) the standard deviation of methylation of individual reads, and iii) the standard deviation of methylation of individual CpG positions. The methylation values of any CpG sites in the flanking sequences (not shown here) have not been considered. The bubble chart depicts the methylation states of each CpG position. Each row represents a different read and each column represents a different CpG position. Filled and open circles indicate methylated and unmethylated cytosines, respectively, whereas the blue circles indicate CpG positions which did not have any or sufficient sequence information. The CpG positions are arranged in the 5′ to 3′ direction of the sequenced region while going from left to right in each read. (**B**) The ‘sprinkler plot’ for the Female #1 brain sample showing the relation of the overall CpG methylation percentage of each sequenced IAP LTR locus to the standard deviation of CpG methylation percentage of individual reads of that locus. Likewise, in (**C**) the relations of the overall CpG methylation percentages of the loci to their respective standard deviations of CpG methylation percentage of individual CpG positions (only the ones sequenced) is shown. In both the sprinkler plots, each dot represents a single IAP LTR locus. Four representative patterns of CpG methylations of the loci have been shown in bubble charts by taking sequencing data from the mentioned sample: (**D**) near-unmethylation, (**E**) read-driven hypomethylation, (**F**) CpG position-driven hypomethylation, and (**G**) near-methylation. The percentages at the bottom of each bubble chart shows their respective overall CpG methylation percentage (M), standard deviation of CpG methylation percentage of individual reads (R), and standard deviation of CpG methylation percentage of individual CpG positions (C). The approximate positions of these representative loci have been indicated in (**A**) and (**B**) by the red, blue, orange, and purple arrows for (**D**), (**E**), (**F**), and (**G**), respectively.

The DNA methylation pattern of a given IAP LTR was characterized with three different values (**Fig. 2A**). These values were derived from the methylation values of the CpGs within the IAP LTR and not from the flanking unique sequences. First, an overall CpG methylation level is the average value that has been calculated from the methylation values of individual sequence reads for a given IAP LTR. Second, a read-based standard deviation measures the deviation level of the methylation value of each sequence read from the overall CpG methylation value. A high value in this standard deviation indicates the presence of potential allele- or cell type-specific DNA methylation for a given IAP LTR. Third, a CpG position-based standard deviation measures the deviation level of the methylation value of each CpG position from the overall CpG methylation value. A high value in this category indicates the presence of potential CpG position-specific DNA methylation. The overall CpG methylation value of each IAP LTR (as a value on X axis) was plotted against either its read-based standard deviation value or CpG position-based standard deviation value (as a value on Y axis). As an example, two plots were generated using the entire set of the sequenced IAP LTR (about 6,500 elements) that had been derived from the brain of Female#1 (**Fig. 2B&C**). Due to the visual similarities of such plots to sprinklers, these plots are referred to as ‘sprinkler plots’ from hereon. A similar series of calculations and plots was also repeated with the other remaining sets of sequence reads (**Fig. S2**).

Inspection of these sprinkler plots of the brain of Female#1 provided the following conclusions. First, the majority of IAP LTR (90%) are positioned within the 80–100% methylation level, consistent with the fact that the majority of retrotransposons are usually repressed by DNA methylation [9]. As shown in **Fig. 2G**, the methylation pattern of this group (80–100% methylation level) is overall similar to each other without any major variations. Interestingly, this group of IAP LTR appears to be distributed differently between two sprinkler plots: loci in this group spread out more upward on the CpG position-based sprinkler plot than on the read-based sprinkler plot (**Fig. 2B&C**). This indicates that the variations observed in the DNA methylation levels of this group likely stem from the methylation differences between CpG positions rather than between sequence reads. Second, a small fraction of IAP LTR (10%) display less than 80% methylation levels. This hypomethylated group can be represented by 4 distinct methylation patterns: near-unmethylation, read-driven hypomethylation, CpG position-driven hypomethylation (**Fig. 2D–F**), and mosaic pattern-driven hypomethylation (not shown). The read- and CpG position-driven hypomethylation patterns are characterized by high values in read- and CpG position-based standard deviation values, respectively. By contrast, the mosaic pattern-driven hypomethylation shows similar values between both types of standard deviation. In summary, the majority (90%) of IAP LTR display a near-complete methylation pattern without any major variation, whereas the minor fraction (10%) displays hypomethylation with various DNA methylation patterns. This trend is also true for all 12 different samples tested in the current study.

### DNA methylation level variations of the IAP LTR retrotransposons

**Figure 3:**
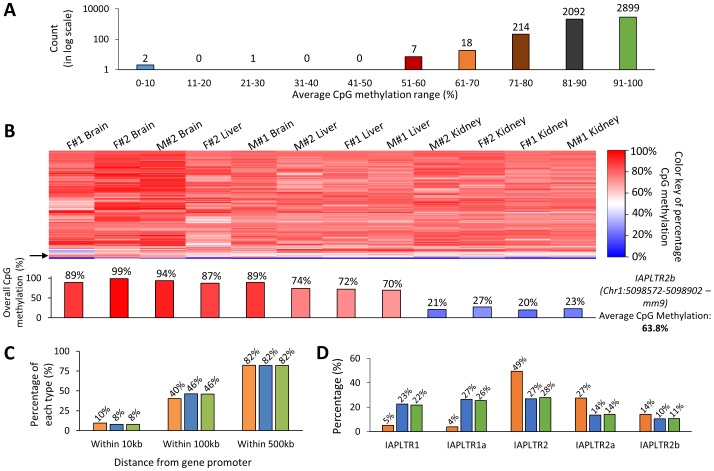
Analyses of the hypomethylated IAP LTRs. (A) A breakdown of the IAP LTRs in the 5,233 representative set based on their average CpG methylation percentage in the twelve samples. The numbers on top of each bar indicates the count of loci in that category. Approximately 5% of the IAP LTR loci (242 loci) have less than 80% CpG methylation levels (hypomethylated). (**B**) Heatmap showing the difference in CpG methylation of the hypomethylated IAP LTR loci (less than 80% CpG methylation levels) among the 12 sequenced samples. The color key on the right depicts the colors representing each value of CpG methylation (blue: 0%, white: 50%, and red: 100%). The tissues in the heatmap have been ordered/clustered based on the differences in CpG methylation of the IAP LTR loci among the tissues (dendrogram not shown). The variation in the CpG methylation among the samples for one particular locus (IAPLTR2b at the position chr1: 5098572–5098902, mm9) is represented by the bar graph below the heatmap. The approximate position of this locus on the heatmap is shown by the black arrow on the left. The bar graph shows the CpG methylation for that locus varies from 20% to 99% among the samples even though the average CpG methylation of that locus is 63.8%. (**C**) The percentage of the hypomethylated (<80%) and mostly methylated (>80%) loci that are within 10, 100, and 500 kb of the promoters of known genes. The orange, blue, and green bars indicate hypomethylated, mostly methylated, and the combined loci (5,233 representative set), respectively. (**D**) Percentage representation of the different subtypes of IAP LTR among the hypomethylated, mostly methylated, and combined categories. Once again the colored bars represent the three categories as mentioned earlier.

The methylation levels of IAP LTR are known to be variable between tissues (intra-individual variation) and also between individuals (inter-individual variation) [14],[15]. A series of systematic analyses were performed to detect potential intra- and inter-individual variations of DNA methylation levels among the sequenced IAP LTRs. We first tabulated together all the methylation values of the sequenced IAP LTRs, which were derived from 12 different samples (**Table S1**). For the subsequent analyses, we retained only those IAP LTR that had been represented by all 12 samples, producing a representative set of 5,233 IAP LTRs. We also calculated average methylation levels and standard deviation values for the 5,233 IAP LTRs using the individual methylation values derived from the 12 different samples. Based on the results from this initial analysis, the representative set could be further divided into the following categories: less than 80%, 80-90%, and 90–100% methylation level categories with 4.6% (242), 40.0% (2,092), and 55.4% (2,899) representation, respectively (**Fig. 3A**).

The methylation levels of the 5,233 IAP LTRs from the 12 samples were clustered and visualized as heatmaps: all loci (**Fig. S3**) and loci with less than 80% methylation level (**Fig. 3B**). Careful inspection revealed the presence of DNA methylation level variations among a large fraction of the representative set. This is particularly obvious among the hypomethylated group (less than 80% methylation level) since the ranges of methylation differences between the samples are much greater than those of the remaining IAP LTRs (greater than 80% methylation). In this group, more than half of the members display a very wide range of DNA methylation levels between the tissues and also between the individuals. One representative locus with 63.8% average methylation level is shown in **Figure 3B**: this IAP LTR shows a range of 20–99% methylation levels among the 12 samples. This group of IAP LTR was further examined to find any features that may be associated with, or responsible for, the highly variable levels of DNA methylation. We examined many features, including genomic locations (**Fig. 3C** **and Fig. S4**) and epigenetic modifications (data not shown), but we have not found any shared features that are closely associated with this group. Interestingly, however, two subtypes (IAPLTR2 and IAPLTR2a) are represented more frequently than expected in this group given their relative compositions in the mouse genome in terms of their numbers compared to the other subtypes (**Fig. 3D**). Furthermore, these two subtypes are not the youngest group among the subtypes. Compared to the youngest group (IAPLTR1), many members of these two subtypes are solo LTRs without the two ORFs (Open Reading Frames), which are essential for their retrotransposition. Thus, frequent hypomethylation on these two subtypes might be an indication for the relaxation of DNA methylation-mediated repression by the host genome.

**Figure 4:**
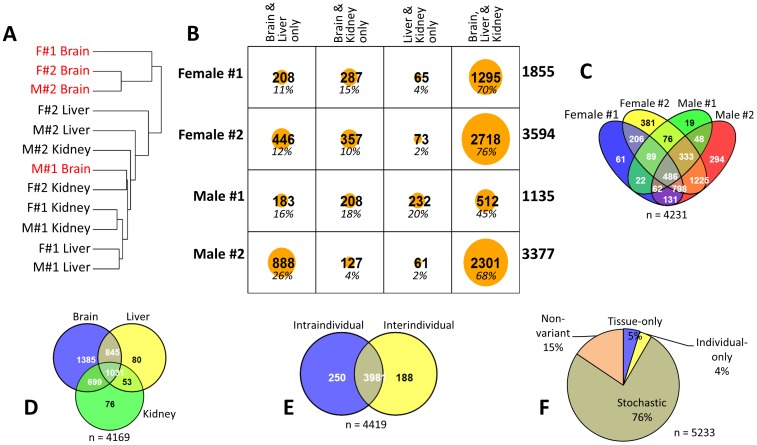
DNA methylation variations of the IAP LTR retrotransposons. (A) Dendrogram showing the clustering of the 12 sequenced samples based on the differences in CpG methylation of the IAP LTR loci. The brain samples tend to show much different CpG methylation than the other samples. (**B**) Intra-individual variation (between tissues of the same individual) of CpG methylation. Matrix showing the numbers of IAP LTR loci that have been found to be statistically significantly different (Kruskal-Wallis test; p<0.001) in their CpG methylation percentages between brain, liver, and kidney in different combinations in the four individuals (Female#1-2, Male#1-2) that have been sequenced. The numbers on the far right of each row indicate the total number of IAP LTR loci that have been found to be varying in that particular individual while the percentages in italics in each cell indicate the number of loci in the respective cells as percentage to the total number of loci for that individual. (**C**) Four-way Venn diagram showing the number of overlapping and non-overlapping IAP LTR loci of the four individuals that have been found to be varying intra-individually. (**D**) Inter-individual variation (between individuals in the same tissue) of CpG methylation. Three-way Venn diagram showing the number of overlapping and non-overlapping IAP LTR loci of the three tissues that have been found to be varying inter-individually (Kruskal-Wallis test; p<0.001). Once again brain samples show more difference in their CpG methylation than the other tissues, since the brain has much higher number of IAP LTR whose CpG methylation varies exclusively in that tissue among the individuals. (**E**) Intersection between IAP LTR loci varying intra-individually and those varying inter-individually. (**F**) Number of loci of the four categories of CpG methylation variation as a percentage of the total number of loci in the representative IAP LTR set.

As described earlier, the remaining IAP LTRs (mostly in the 70–100% average methylation range) also display inter- and intra-individual variations in their DNA methylation levels, but the ranges of these variations are much narrower (10–30% differences) than those of the hypomethylated group. Thus, to confirm the statistical significance of these variations, the entire representative set including the hypomethylated group was analyzed again using a series of statistical tests (Kruskal-Wallis test; p<0.001; **Materials & Methods**). For the intra-individual variations, a total of 4,231 IAP LTR displayed statistically significant variations in the DNA methylation level: the number of variable IAP LTRs ranged from 1,135 to 3,594 among the four individuals (**Fig. 4B&C**). Detailed inspection further revealed that the majority of these loci showed variations in all three tissues. Interestingly, the number of IAP LTR varying only between the liver and kidney is the least among all combinations of the two-tissue comparison. This may be an indication that the IAP LTR has a much different CpG methylation status in the brain than in the two other tissues. Such assumption is supported by the clustering of the 12 samples based on their CpG methylation values of the 5,233 loci, where three out of the four brain samples were much closer to each other but much further than the other samples (**Fig. 4A**; the brain samples marked in red). For the inter-individual variations, a total of 4,169 IAP LTR were found to have statistically significant variations at least in one of the tissues examined among the four individuals (**Fig. 4D**). Even in this group, the brain also showed the most difference (3,960) while the liver and kidney showed variations at the 2,009 and 1,859 loci, respectively. These two groups of IAP LTR with intra- and inter-individual variations were further compared to each other. According to the results, a total of 4,419 loci showed variations in at least one combination of the comparisons (**Fig. 4E**), whereas the remaining 814 loci did not show any statistically significant variations – the non-variants (**Fig. 4F**). Overall, this series of statistical analyses identified four categories of IAP LTR: i) Tissue-only variable loci (varying intra-individually only), ii) Individual-only variable loci (varying inter-individually only, also defined as epialleles) [29], iii) Stochastically variable loci (varying both intra- and inter-individually), and iv) Non-variant loci. These four categories of IAP LTR are presented in **Table S1**. In sum, a series of analyses concluded that majority of the IAP LTR (85%) show variations in the DNA methylation levels, intra- and/or inter-individually. Also, the DNA methylation variations of IAP LTR are more prevalent in the brain than in the other two tissues examined, kidney and liver.

## Discussion

In this study, we tested the feasibility of a newly developed high throughput bisulfite sequencing protocol termed HT-TREBS. With this protocol, we were able to analyze the DNA methylation status of a large number of IAP LTRs, representing more than 80% of the 5 subtypes of this retrotransposon family. The results indicated that a small subset of these repeat elements are hypomethylated with their average DNA methylation levels being less than 80% across the twelve samples. Majority of IAP LTRs are also variable in terms of their DNA methylation levels, intra- and/or inter-individually. Overall, the current study demonstrates the effectiveness of HT-TREBS, and also provides insights regarding the patterns and levels of DNA methylation of the IAP LTR retrotransposon family.

One of main strengths of HT-TREBS is to measure the DNA methylation levels of highly repetitive DNA sequences not only on an individual locus basis but also in a high throughput manner. This has not been easy for other genome-wide DNA methylation analyses because mapping of short sequence reads with repetitive sequences has not been feasible until now. To solve this problem, HT-TREBS is designed to target a large number of genomic loci encompassing both repeat and adjacent non-repeat regions. The non-repeat regions are subsequently used for mapping of their associated repetitive sequences to individual genomic loci. The actual scheme of HT-TREBS employs a semi-specific PCR strategy in which one primer binds to a repeat region while the other primer binds to an adaptor that is added for library construction (**Fig. 1A**). According to the raw statistics from our sequencing runs, this targeting has been very efficient: in any given sample, about half of the 6–8 million sequence reads were IAP LTR sequences (data not shown). With one round of a semi-specific PCR, this level of enrichment (about 50% success rate) appears to be very robust given the fact that many previous attempts to enrich repeat families with similar PCR schemes have been rather inefficient. In the case of HT-TREBS, this may have been possible mainly due to a unique feature of the added adaptor, which contains all the Cs as methylated Cs. The PCR primer binding to this methylated adaptor probably had a high level of selection power since the majority of Cs without methylation in the genomic DNA had already been converted into Us (later into Ts) by the bisulfite conversion reaction. Nevertheless, HT-TREBS also needs further improvements since the mapping of the sequenced IAP LTR was somewhat ineffective: only approximately 1–2 million out of 3–4 million IAP LTR sequence reads were properly mapped to individual genomic loci. This may have been caused by relatively short lengths of the non-repeat regions within individual IAP LTR reads. In the initial scheme of HT-TREBS, the size selection step had to enrich DNA fragments with less than 300 bp in length mainly because the available NGS machines had a limited read length (at longest, 300 bp). In retrospect, this may not have provided sufficient lengths of the non-repeat regions for the mapping of the sequenced IAP LTR. Thus, one obvious improvement should be utilizing NGS platforms with longer read lengths, which would definitely increase the mapping efficiency of the sequence reads from NGS platforms.

According to the results presented in **Fig. 2**, a small fraction of IAP LTR have less than 80% DNA methylation, and their methylation patterns are represented with 4 different types: near unmethylation, read-driven, CpG position-driven, and mosaic pattern-driven hypomethylation. These 4 methylation types provide some hints regarding how and why these methylation types have been formed on the IAP LTR. First, the IAP LTR with the near-unmethylation type tend to be located in close proximity to the genomic loci with active histone marks, such as H3K4me1 or H3K27ac, although the number of this category is too small to be generalized, less than 10 [30]. These two histone marks are associated with regulatory regions for actively transcribed genes. In contrast, the majority of IAP LTR are usually repressed by H3K9me3 [4],[5]. Given the fact that many LTRs become alternative promoters for the adjacent genes [14],[15],[31], it is reasonable to predict that these IAP LTR might also have become part of the regulatory regions for transcription of the adjacent genes. Second, the read-driven hypomethylation is likely caused by either allele-specific or cell type-specific DNA methylation on IAP LTR. If an IAP LTR is subject to allele-specific DNA methylation, the overall methylation level should be 50% as seen in imprinted genes [32]. On the other hand, if the methylation pattern is caused by the different levels of DNA methylation between individual cell types, the average methylation levels should fluctuate among the individual DNA samples since each organ, such as brain, kidney, and liver, should have different proportions of individual cell types. Third, a very small fraction of IAP LTR belongs to the CpG position-driven hypomethylation type. In these IAP LTR, interestingly, the unmethylated CpGs are usually positioned at the boundaries between the non-repeat and IAP LTR regions. This might be an indication that IAP LTR also starts losing their DNA methylation from the boundary regions (shores) as seen in CpG islands [33]. Fourth, the mosaic pattern of DNA methylation might be caused by the accumulation of independent mistakes of DNA methylation maintenance during DNA replication. DNMT1 is known to have a 5% error rate during DNA replication [34], thus this is a likely cause for this type of hypomethylation. The mosaic patterns observed in the IAP LTR are very random without any shared patterns, probably reflecting the random nature of mistakes by DNA methylation machineries. Taken together, although this group of IAP LTR shares a common feature, DNA hypomethylation, the four distinct patterns clearly indicate quite different paths for the formation of DNA methylation on these IAP LTR.

IAP LTR has been known to be variable in terms of their DNA methylation levels (17). The current study further strengthens this initial observation by providing the total numbers and actual locations of individual IAP LTR with DNA methylation variations. The current study also derived two additional findings regarding the DNA methylation variations of IAP LTR. First, the relatively large number of IAP LTR are shown to have DNA methylation level variations, intra- and/or inter-individually. The estimated number of variable IAP LTR could be easily more than 80% of the entire tested IAP LTR (**Fig. 4**). This estimate is somewhat surprising, but it is also possible given the evolutionary age of IAP LTR: the majority of IAP LTR are thought to have retrotransposed into the mouse genome in a very recent evolutionary time [35]. Thus, it is reasonable to think that implementing the DNA methylation-mediated repression on these newly inserted DNA may be incomplete and still in progress, thus resulting in intra- and inter-individual DNA methylation variations among a large number of individual IAP LTR. Second, the DNA methylation level variation of IAP LTR is more prevalent in the brain than in the other two organs, kidney and liver (**Fig. 4**). This observation may be reflecting the fact that brain is made up of a greater number of cell types than the other tissues. Each cell type is thought to have a different epigenome, thus it is likely that the mammalian brain with a greater diversity of cell types may have greater variations in DNA methylation of the retrotransposon family. According to the recent results from humans, another retrotransposon family, L1, may be responsible for cellular mosaicism in neuronal cells via retrotransposition, which may in turn contribute to the increase of the cellular diversity in human brains [36],[37]. If this is also true for the other retrotransposon families, the high levels of the DNA methylation variations observed from IAP LTR may also be reflecting these unknown roles in brain. Overall, the DNA methylation level of IAP LTR is highly variable intra- and inter-individually, and this variability is more prevalent in the brain than the other organs of the mouse with unknown reasons, which requires further investigation in the near future.

## Materials and Methods

### Ethics Statement

All the experiments related to mice were performed in accordance with National Institutes of Health guidelines for care and use of animals, and also approved by the Louisiana State University Institutional Animal Care and Use Committee (IACUC), protocol #10-071.

### Library construction for HT-TREBS

Genomic DNA has been isolated from the brain, liver and kidney of four 1-week-old littermates (two females and two males) of the C57BL/6N mouse strain (Jackson Lab). For each of the twelve samples, 1 µg of the isolated genomic DNA was sonicated to fragments with their average size being 700 bp in length (Bioruptor NGS, Diagenode). Since the average lengths of IAP LTR are 300-350 bp in length and the desired sequences need to contain non-repeat flanking sequences, the optimum size of the fragments are empirically determined to be around 700 bp in length. The fragmented DNA was immediately end-repaired using the NEB Next End Repair Module (New England BioLabs), and then ligated to custom-made duplex Ion Torrent ‘A’ adaptors using T4 DNA ligase (New England BioLabs). In these adaptors, all the Cs have been methylated (Integrated DNA Technologies). The adaptor-ligated DNA fragments were size-selected to remove any fragment smaller than 300 bp in length using the Agencourt AMPure XP beads (Beckman Coulter). The size and quantity of the selected fragments were analyzed using the Agilent 2100 Bioanalyzer. The adaptor-ligated DNA library was modified using the bisulfite conversion reaction according to the manufacturer's protocol (EZ DNA methylation kit, Zymo Research). The bisulfite-converted ‘A’ adaptor-ligated library was used as a template for a round of PCR (Maxime PCR Premix Kit, Intron Biotech). In this PCR, the forward primer (CCATCTCATCCCTGCGTGTCTCCGACTCAG) was designed to bind to the 5′-end of the ‘A’ adaptor region whereas the reverse primer was designed to bind to the 24-bp conserved region among the consensus sequences of the IAP LTR subtypes (IAPLTR1, IAPLTR1a, IAPLTR2, IAPLTR2a, and IAPLTR2b). The 5′-end of the reverse primer also had the sequence of the regular Ion Torrent ‘P1’ adaptor (CCACTACGCCTCCGCTTTCCTCTCTATGGGCAGTCGGTGAT∧CTCCCTAATTAACTACAACCCATC). The break in the reverse primer sequence indicates the joining point of the P1 adaptor sequence and the IAP LTR-specific sequence. The amplification cycle number of PCR was individually determined for each of the twelve samples so that the minimum possible cycles were used to generate just enough the amount of the PCR product for sequencing. The PCR product was size-selected for a range of 250–300 bp in length using agarose gel electrophoresis. The size-selected PCR product was verified for its length and quantity using the Agilent 2100 Bioanalyzer. Finally, each of the twelve PCR products was individually sequenced in the Ion Personal Genome Machine (PGM) Sequencer using Ion 318 Chips (Ion Torrent, Life Technologies).

### Mapping of the sequences and other computational analyses

The sequence reads generated from the twelve Ion PGM runs were individually mapped to a curated reference genome using the aligner Bowtie 2 [27]. This curated genome is made up of either the bisulfite-converted top (Original Top: OT) or the bottom (Original Bottom: OB) strands of the IAP LTR sequences (along with the 350-bp flanking sequences) depending on the orientation of the specific IAP LTR locus in the reference genome (mm9). The mapped reads were then filtered using custom Python scripts to extract only the sequences that had been mapped to the IAP LTR regions and also had at least 10 bases of the flanking unique sequences to ensure the reads were generated from a specific IAP LTR locus. The filtered reads from each sample were separately analyzed using the BiQ Analyzer HT tool [28] to derive the CpG methylation status of each IAP LTR locus and also other relevant information regarding the quality of the reads. These methylation data were analyzed further with the following filter using custom scripts: i) discard any IAP LTR loci that did not have at least 3 CpG positions for which the sequencing depth was at least 15X, ii) discard any IAP LTR loci that have not been sequenced in all twelve samples. Application of all these filters helped us build the representative set of 5,233 IAP LTR loci. All dataset (raw and processed) of the sequenced samples have been added to the NCBI GEO data repository (http://www.ncbi.nlm.nih.gov/geo/query/acc.cgi?acc=GSE49222).

### Statistical analyses for CpG methylation level variations

The methylation level variations among the representative IAP LTR loci were calculated using the following combinations: i) intra-individual variation (separately in each of the four individuals using the respective brain, liver, and kidney sample methylation data), and ii) inter-individual variation (separately for each of the three tissues using the methylation data of the four individual in the respective tissues). For each specific locus, the CpG methylation values of the reads of the relevant samples were compared with each other to find variations by using the non-parametric Kruskal-Wallis test (p<0.001). Each positive test of variation was followed by a post hoc Mann-Whitney test with Bonferroni corrections to isolate the pair of samples that actually gave rise to the variation.

## Supporting Information

Figure S1 **The genome graphs of the twelve sequenced samples as visualized using the UCSC Genome Browser website shows that the sequenced IAP LTRs are distributed over the entire lengths of all the chromosomes.**
(TIF)

Figure S2 **The two-dimensional read-based and CpG position-based sprinkler plots of the all the twelve samples sequenced (brain, liver, and kidney of Female#1, Female#2, Male#1, and Male#2).** The sprinkler plots have been described in **Figure 2**.
(TIF)

Figure S3 **Heatmap of the 12 sequenced samples showing the difference in CpG methylation of all the representative IAP LTR loci.** The dendrogram on top shows the clustering of the samples while that on the left shows the clustering of individual loci based on their CpG methylation difference. The color key on the top left depicts the colors representing each value of CpG methylation while the histogram in it shows the number of loci present in the heatmap at those respective methylation values.
(TIF)

Figure S4 **(A) A plot of the average methylation of the 5,233 representative IAP LTR loci against their distance from the nearest transcription start site (TSS).** (**B**) A plot of the average methylation of the representative IAP LTR loci against their absolute distance from the nearest TSS. A very low coefficient of determination (*R^2^*) indicates the absence of any particular relation between the distance of the IAP LTR elements and their methylation status.
(TIF)

Table S1 **This supplemental data contains table a through g. Lists of IAP LTRs with detailed information regarding genomic positions, DNA methylation levels derived from 12 samples using HT-TREBS, and their inter and intra methylation variation patterns based on statistical analyses.** (**1a**, Representative-Set; **1b**, Hypomethylated <80%; **1c**, Mostly-methylated > = 80%; **1d**, Tissue-Only-Variation; **1e**, Individual-Only-Variation; **1f**, Both-Intra-Inter-Variation, Stochastically variable; **1g**, Nonvariant).
(XLS)
