# Supplementary figures and images for: High-Throughput Targeted Repeat Element Bisulfite Sequencing (HT-TREBS): Genome-Wide DNA Methylation Analysis of IAP LTR Retrotransposon
Source: PLoS One. 2014 Jul 8;9(7):e101683. doi: 10.1371/journal.pone.0101683 (PMC4086960; doi:10.1371/journal.pone.0101683)

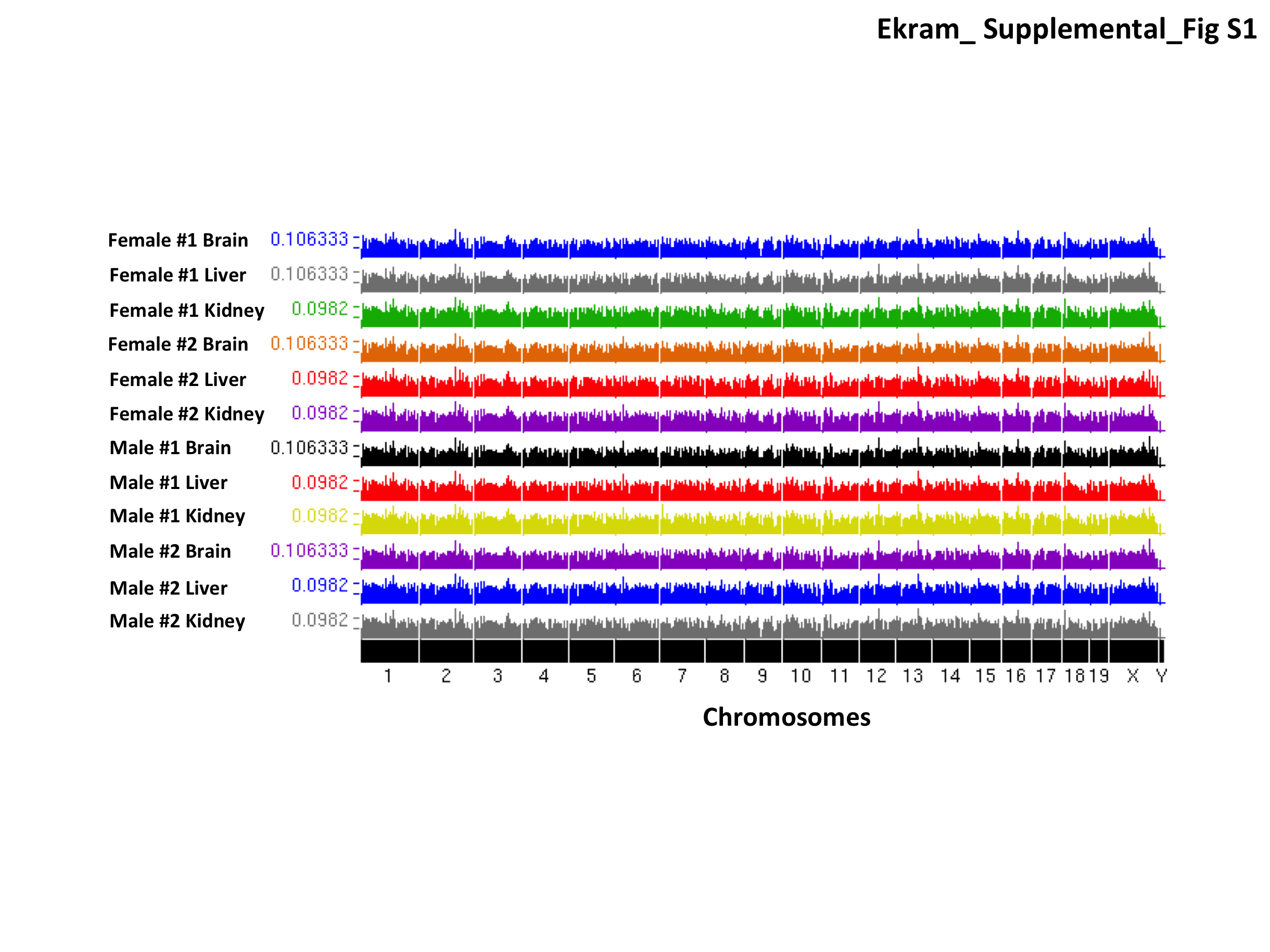

Supplement: Figure S1 — The genome graphs of the twelve sequenced samples as visualized using the UCSC Genome Browser website shows that the sequenced IAP LTRs are distributed over the entire lengths of all the chromosomes. (TIF) [file pone.0101683.s001.tif]

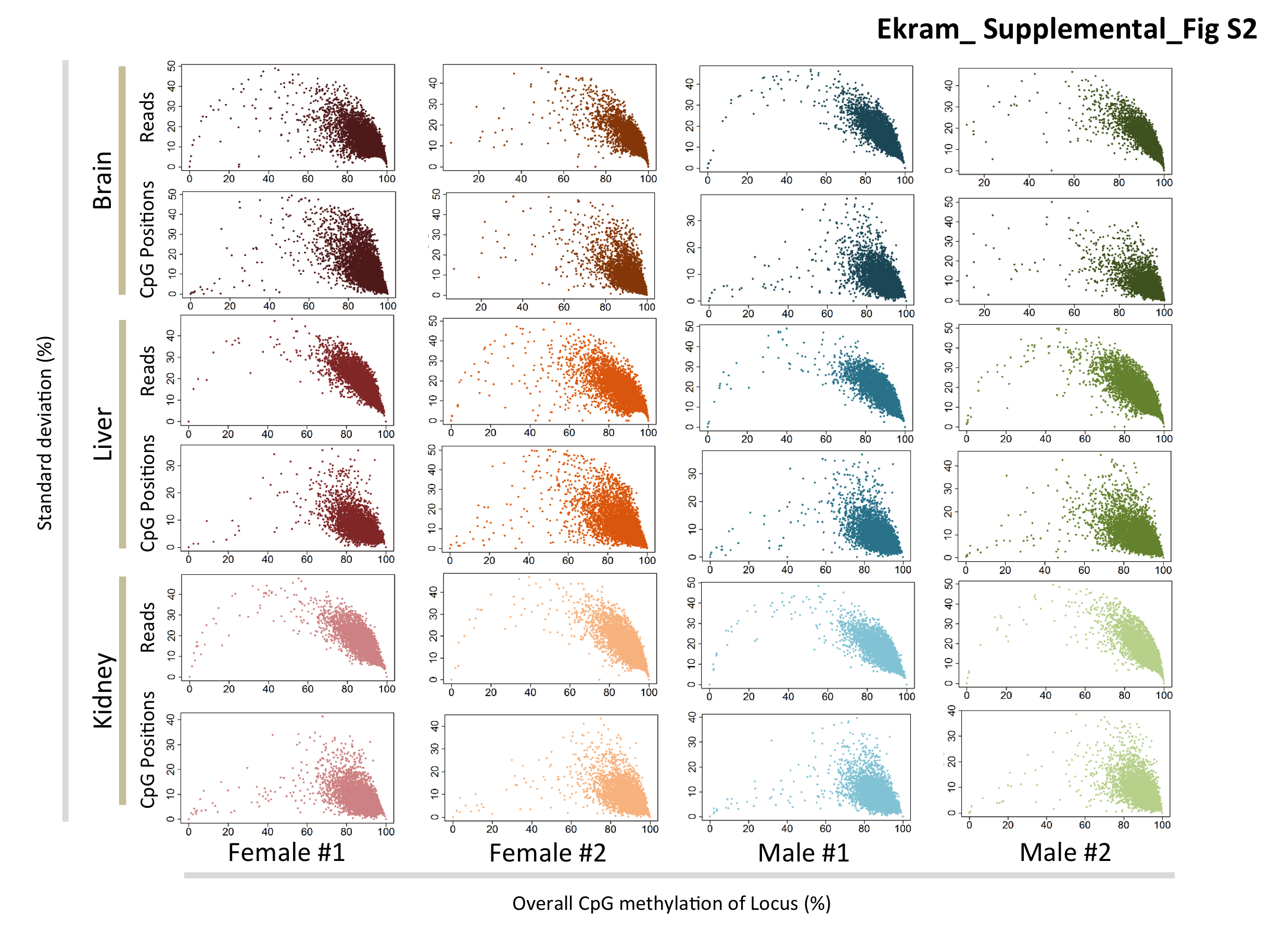

Supplement: Figure S2 — The two-dimensional read-based and CpG position-based sprinkler plots of the all the twelve samples sequenced (brain, liver, and kidney of Female#1, Female#2, Male#1, and Male#2). The sprinkler plots have been described in Figure 2 . (TIF) [file pone.0101683.s002.tif]

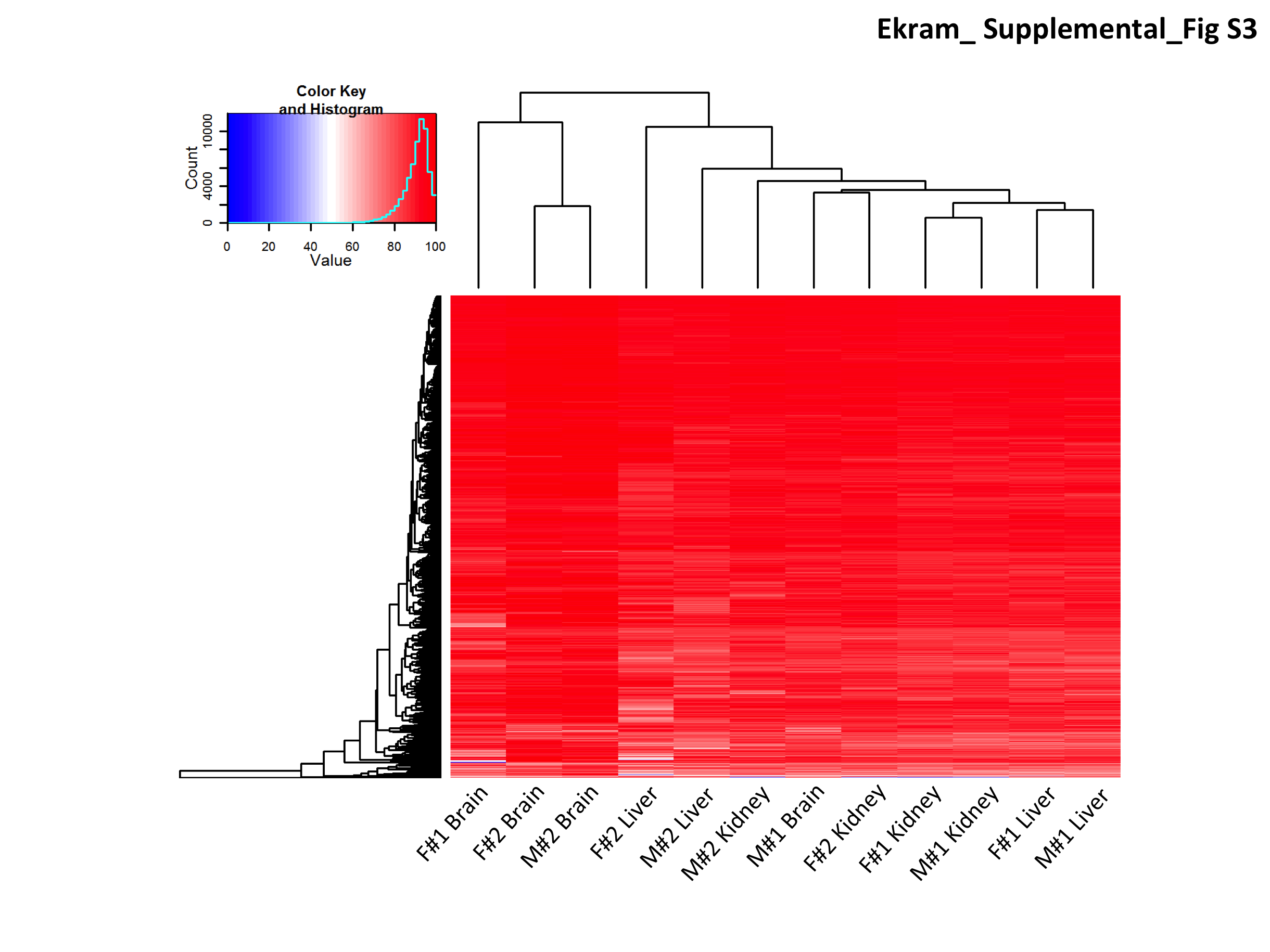

Supplement: Figure S3 — Heatmap of the 12 sequenced samples showing the difference in CpG methylation of all the representative IAP LTR loci. The dendrogram on top shows the clustering of the samples while that on the left shows the clustering of individual loci based on their CpG methylation difference. The color key on the top left depicts the colors representing each value of CpG methylation while the histogram in it shows the number of loci present in the heatmap at those respective methylation values. (TIF) [file pone.0101683.s003.tif]

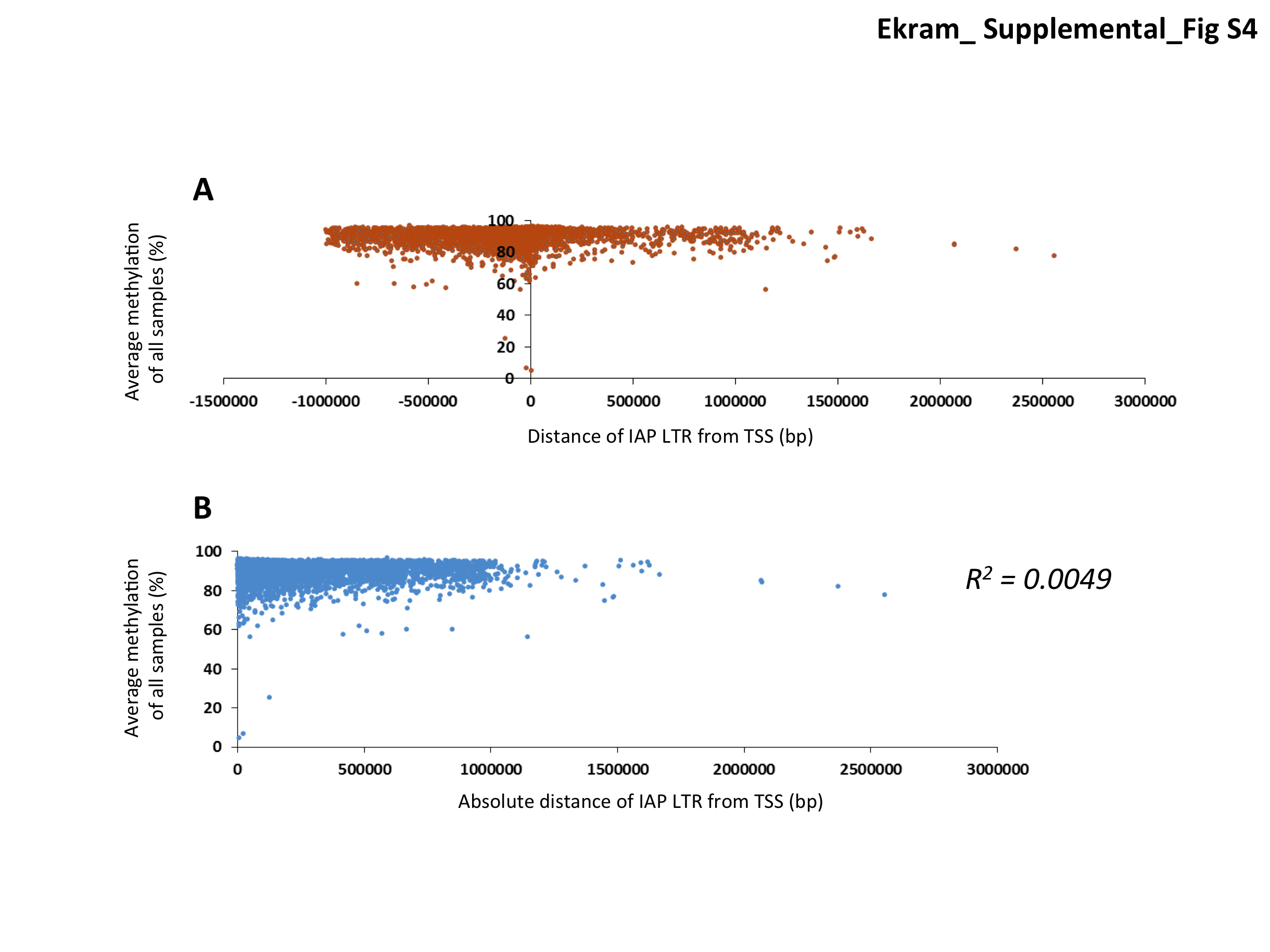

Supplement: Figure S4 — (A) A plot of the average methylation of the 5,233 representative IAP LTR loci against their distance from the nearest transcription start site (TSS). (B) A plot of the average methylation of the representative IAP LTR loci against their absolute distance from the nearest TSS. A very low coefficient of determination (R2) indicates the absence of any particular relation between the distance of the IAP LTR elements and their methylation status. (TIF) [file pone.0101683.s004.tif]
